# Genotypic Characterization of *Orientia tsutsugamushi* Isolated From Acute Encephalitis Syndrome and Acute Febrile Illness Cases in the Gorakhpur Area, Uttar Pradesh, India

**DOI:** 10.3389/fmicb.2022.910757

**Published:** 2022-07-05

**Authors:** Nikita Nanaware, Dipen Desai, Anwesha Banerjee, Kamran Zaman, Mahim Mittal, Mahima Mittal, Smita Kulkarni

**Affiliations:** ^1^Indian Council of Medical Research (ICMR)–National AIDS Research Institute, Pune, India; ^2^Indian Council of Medical Research (ICMR)–Regional Medical Research Centre, Gorakhpur, India; ^3^Baba Raghav Das (B.R.D.) Medical College, Gorakhpur, India

**Keywords:** genotypic characterization, *Orientia tsutsugamushi*, Acute Encephalitis Syndrome, scrub typhus, dual infection, Gorakhpur, India

## Abstract

Scrub typhus infections caused by *Orientiatsutsugamushi* (OT), continue to remain underdiagnosed globally, due to the lack of distinctive symptoms. The elusive nature of the Acute Encephalitis Syndrome (AES) outbreak in Gorakhpur, Uttar Pradesh that claimed numerous pediatric lives was the driving force of this study which involved serological diagnosis (IgM–ELISA), isolation of OT in cell culture, confirmation by PCR, and characterization by Sanger sequencing. In total, 12 out of 36 patients were seropositive, of which 4 were positive by PCR. Upon enrichment in cell culture, additional 3 patients (including two seronegative) were detected positive by PCR. In total, three of these 7 patients were found to be infected with two strains of OT. Taken together, this study for the first time reports the occurrence of dual infections in addition to three circulating OT genotypes (Gilliam, Kato, and Karp-like) and highlights the significance of enriching OT in cell culture systems for efficient molecular detection.

## Introduction

Scrub typhus (ST), a vector-borne disease, caused by *Orientia tsutsugamushi* (OT) is a, neglected tropical disease. This disease was initially confined to the tsutsugamushi triangle, however, in recent times the disease has been reported from other parts of the world (Banerjee and Kulkarni, 2021). Among the rickettsial infections such as Rocky Mountain spotted fever, Epidemic typhus, Q fever, etc., ST is more commonly reported in various states of India (Rahi et al., 2015).

OT, the Gram negative obligate intracellular bacteria are suspected to be one of the causes of Acute Encephalitis Syndrome (AES) and Acute Febrile Illness (AFI) that often remain underdiagnosed since the initial symptoms are very common and similar to that of flu or any other viral infection (Wongsantichon et al., 2020). Also, the lack of the pathognomonic eschars, characteristic of ST, adds to the misdiagnosis (Kundavaram et al., 2013; Prakash, 2017). Although a well-timed treatment with doxycycline helps in the speedy recovery of the patient in initial phases of infection, lack of medical attention or delay in treatment may lead to severe complications such as multiple organ failure that may be lethal (Rapsang and Bhattacharyya, 2013; Koraluru et al., 2017).

The outbreak of AES in Gorakhpur, Uttar Pradesh (UP) that sought global attention due to the enormous loss of pediatric lives was predominantly due to the Japanese Encephalitis (JE) virus. Subsequently, vaccination for JE was undertaken which reduced JE infections; however, the incidence of AFI with neurological manifestations persisted (Kumari and Joshi, 2012; Mittal et al., 2017). Investigations addressing the issue of AES of unknown etiology concluded re-emergence of ST in many reports from Gorakhpur. A study conducted in September–October 2015 (Murhekar et al., 2016) in children with AES and AFI reported the presence of IgM antibodies, respectively, in 63 and 54% children, suggesting the role of ST in the etiology of AES in the Gorakhpur region. However, the low PCR positivity rate (1.6 and 3.7%, respectively), negligible occurrence of the classical eschar and minimal or no response to the standard treatment (Azithromycin) raised doubts about the ST diagnosis. A subsequent study found out the existence of antibodies against OT during AES epidemic period (July–November 2016) as well as during the lean period (December–June 2016), antibody titer being higher during the epidemic period (Kamble et al., 2020). Apart from these studies, various other reports were based on either serological or molecular evidences but none involved isolation of pathogen in cell culture that is a definite method for diagnosis of any disease of unknown etiology (Janardhanan et al., 2014; Varghese et al., 2015; Jain et al., 2017; Chunchanur et al., 2019; Kumar et al., 2019; Gupte et al., 2020; Kannan et al., 2020).

Understanding the magnitude of the outbreaks, gaps in the knowledge and unavailability of adequate facilities for confirming the causative agent were some of the road blocks in the disease management and increasing the fatality in ST infections. The manifestations of ST are not unique and is the major reason for its misdiagnosis, the second being the larger variability among the strains. Therefore, constant advancements in the field of OT diagnostics are being made with the application of ELISA, PCR and immunostaining techniques. In context with the aforementioned reasons, a study that aimed at isolation and characterization of the causative agent of few AES and AFI cases reported in Gorakhpur in 2018 was undertaken which also facilitated re-establishment of a dedicated and one of its own kinds Rickettsiology Laboratory in India that holds the potential to handle seasonal outbreaks in terms of diagnosis and research.

## Methodology

### Cell Lines and Bacterial Stocks

Standard strains of OT (Karp, Kato, Kostival, Gilliam) and *Rickettsia* spp. (*R. conori*, *R. akari*, *R. Rickettsiiand*, and *R. typhi*) along with cell line L-929 were procured from the Rickettsial Reference Laboratory, CDC, Atlanta, United States. The cell lines were maintained in Gibco’s Minimum Essential Medium (MEM), supplemented with 5% FBS (heat inactivated), L-glutamine, non-essential amino acids and HEPES (complete MEM) and incubated with 5% CO_2_ at 37°C. The stocks of the standard strains were developed in L-929 and Vero-E6 cell lines and used for the optimization of the isolation and PCR protocols as per previous reports (Enatsu et al., 1999; Zhang et al., 2013; Masakhwe et al., 2018; Kannan et al., 2020).

### Clinical Specimen Collection and Storage

In total, thirty-six patients suffering from AES and AFI cases residing in the six districts (Gorakhpur, Kushinagar, Basti, Siddharth Nagar, Maharajganj, and Deoria) of UP and one district (Gopalganj) of Bihar were enrolled in the study during the epidemic period (15 July to 3 August 2018) at the BRD Medical College, Gorakhpur, UP. In total, 49 clinical specimens which included 13 paired cerebrospinal fluid (CSF) and blood clots from AES pediatric patients, only blood clot specimens from 15 pediatric AFI cases and 8 whole blood specimens from adult AFI cases were collected. The pediatric cases were tested for IgM ELISA for OT at the site of collection. The CSF and whole blood specimens were stored at –80°C while blood clots were stored at 4°C until further processing. All the patients were treated as per the institutional AES/AFI treatment protocol. Patients with AES were given intravenous azithromycin and patients with AFI were given oral azithromycin/doxycycline. All the patients except one recovered and were discharged from the hospital.

### Enrichment of Clinical Specimens

The CSF was diluted (1:5) in the complete MEM before infection. Blood clots were disintegrated by pulse vortexing with sterile glass beads, after which samples were briefly centrifuged and supernatant was used for infection. Whole blood specimens were directly used for infection. The processed specimens were overlaid on the monolayer of confluent L-929 cells in a tissue culture flask having surface area of 25 cm^3^ (T-25). The infected cells were observed microscopically daily for the cytopathic effect (CPE). Irrespective of the CPE, the cells were harvested by scraping the cells from a small area in T-25 flask at multiple time intervals such as 3, 5, 7, and 10 post-infection days (PIDs), followed by blind passage on 10th and 20th PID. All the samples were harvested, aliquoted, and stored in the freezing medium (complete MEM + 5% FBS + 10% DMSO) on the 30th PID. Regardless of the serological or molecular results, all the samples were cultured in cell lines.

### Molecular Detection and Phylogenetic Analysis of the Cultured Specimens

DNA from the clinical specimens and at various time intervals post-culturing was extracted using QIAGEN Blood DNA extraction kit (Qiagen, United States). Primary detection of OT was carried out using primer sets specific toward: (a) groESL gene encoding heat-shock protein and (b) 56 kDa outer membrane protein, also known as type specific antigen (TSA). The sequences of the primer are given in Table 1 of the supplementary data. Two sets of primers covering 3 of 4 hyper variable regions in 56 kDa gene were further used for confirmation of OT by nested PCR and phylogenetic analysis by Sanger sequencing. The sequences of the primers used and the reaction conditions for PCR are mentioned in Table 1. The reaction chemistry used for PCR comprised of 2X platinum hot-start master-mix (Invitrogen, Themo Fischer Scientific, United States).

**TABLE 1:** Primers and conditions used for PCR and sequencing.

Primer		Sequence	Reaction conditions
Round I	A	TTTCGAACGTGTCTTTAAGC	Initial Denaturation: 94°C, 2 min Denaturation: 94°C, 1 min Annealing: 57°C, 1:20 No. of cycles: 30 Extension: 72°C, 2 min Final extension: 72°C, 7 min
	B	ACAGATGCACTATTAGGCAA	
Round II*	C	GTTGGAGGAATGATTACTGG	Initial Denaturation: 94°C, 2 min Denaturation: 94°C,1 min Annealing: 55°C, 45 s No. of cycles: 25 Extension: 72°C, 1.20 min Final extension: 72°C, 5 min
	D	AGCGCTAGGTTTATTAGCAT	

*Amplified product from the Round I PCR was used as a template in the round II PCR.

Furthermore, the PCR-amplified product was subjected to agarose (2%) gel electrophoresis using SyBr Safe stain to confirm presence of OT. The images of the gel were captured using EC3 Imaging System (UVP Bio imaging systems, Canada). The round II PCR amplified product was purified from agarose gel. Since there was loss of DNA after gel extraction, the purified fragment was re-amplified by using round II PCR primers. The purity of re-amplified DNA was verified on gel and further used as template for sequencing. Sanger sequencing was carried out using the same primer pair (round II) and Big Dye terminator v 3.0 chemistry on ABI 3,730xl DNA Analyzer (Applied Biosystems, Thermo Fisher Scientific, United States).

The sequences amplified by each, forward and reverse primer were aligned in MEGA 7.03 software. The sequences aligned were cured, trimmed manually to achieve a uniform length, and the phylogenetic relationship was established using software MEGA 7.03 by maximum likelihood keeping the bootstrap value 1,000.

## Results

### Serological Investigations

On site serological investigations carried out on 35 out of 36 enrolled patients (one diagnosed clinically) at the BRD Medical College, Gorakhpur detected 12 patients positive for ST by IgM ELISA. The results of the serological testing are given in Table 2.

**TABLE 2:** Molecular detection of samples positive for OT (pre- and post-enrichment) along with the serological results.

Sr. No.	Sample ID	AES/AFI	OT-IgM ELISA	Sample type	Positivity by nested PCR		
					Pre-enrichment	Post-enrichment	At nth PID
1.	NARI-01	AES	+	CSF	–	+	3
				Blood clot	+	+	7
2.	NAR-02	AES	+	CSF	–	+	3
				Blood clot	+	+	7
3.	NARI-03	AES	+	Blood clot	+	–	–
4.	NARI-06	AES	–	CSF	+	+	6
				Blood clot	–	+	7
5.	NARI-07	AES	–	CSF	–	+	3
6.	NARI-26	AFI	+	Blood clot	+	+	20
7.	NARI-33	AFI	+	Whole blood	+	+	20

### Molecular Detection of the Cultured Specimens

As most of the patients manifested the symptoms of ST but were negative by IgM ELISA, attempts were made to enrich the bacteria in cell lines so that it can be detected by molecular techniques. All CSF, blood clot, and whole blood specimens were tested by nested PCR targeting the 56 kDa gene, pre- and post-culturing in L-929 cell line. Initially, OT could be detected in six samples: one CSF (NARI-6), three blood clot (NARI-1, NARI- 2, NARI- 3) specimens from AES cases, and one blood clot (NARI-26), one whole blood (NARI-33) specimens from AFI cases. After culturing in L-929 cells, OT was detected in nine samples: four CSF (NARI-1, NARI-2, NARI-6, and NARI-7), four blood clots (NARI-1, NARI-2, NARI-6, and NARI-26), and one whole blood (NARI-33) specimens. In total, two patients (NARI-6 and NARI-7) who were reported negative in IgM ELISA for OT were found to be positive by PCR post-culturing in L-929. Also, the CSF specimen from one patient (NARI-6) was found to be positive in OT-PCR pre- as well as post-enrichment despite being reported sero-negative. Table 2 summarizes the details of ST detection by both, serological and molecular methods.

### Phylogenetic Analysis of the Detected *Orientia tsutsugamushi* Strains

**FIGURE 1:**
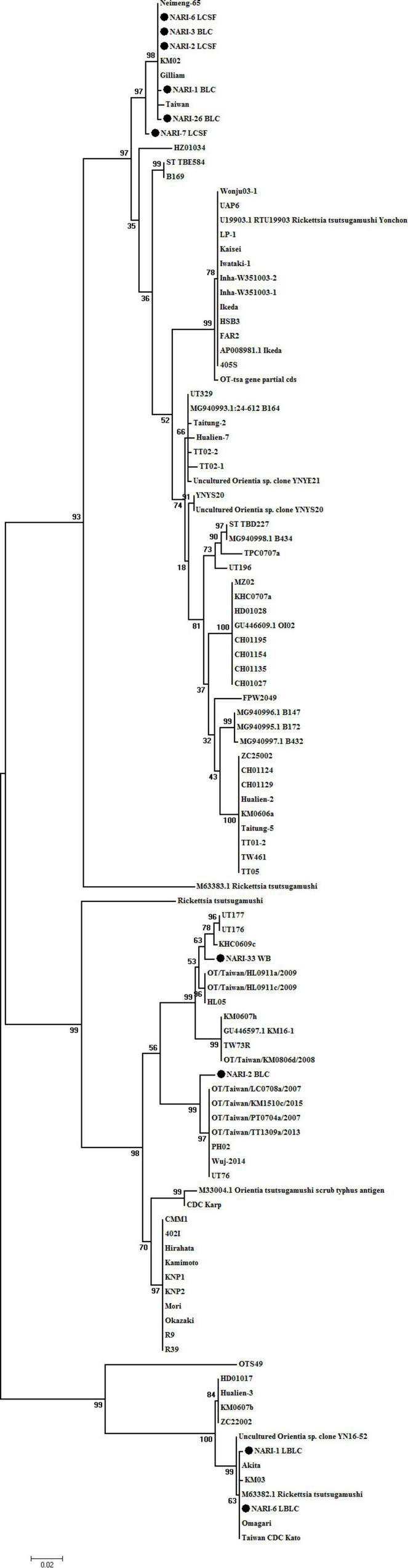
Phylogenetic tree of *O. tsutsugamushi* strains constructed based on base-sequence homologies of 56 kDa type-specific genes. The numbers at nodes indicate bootstrap values. Bar shows genetic distance of 0.02. The isolates of from the study are submitted to GenBank under accession numbers as follows: accession numbers of published sequences: OTNARI-1_BLC (OK019092), OT NARI-3 BLC (OK019093), OT NARI-26 BLC (OK019094), OT NARI-1 LBLC (OK019095), OT NARI-2 BLC (OK019096), OT NARI-2 LCSF (OK019097), OT NARI-6 LCSF (OK019098), OT NARI-6 LBLC (OK019099), OT NARI-7 LCSF (OK019100), and OT NARI-33 WB (OK019101).

PCR confirmed ST in samples from 7 patients. Isolation of OT from the CSF, blood clot, whole blood specimens and sequencing of specimens pre- and post-enrichment revealed 10 different sequences clustering with Gilliam, Kato, Karp-like clinical isolate (Figure 1). The pre- and post-cultured samples that originated from an individual that had identical 56 kDa gene sequences were submitted as a single sequence in GenBank. The samples that had distinct nucleotide sequences despite of sharing the same origin (specimens collected from the same patient) have been submitted to the GenBank separately. Prevalence of three genotypes of OT: Gilliam, Kato, and Karp-like was detected of which Gilliam strain was predominantly found in most of the samples (Figure 1).

Briefly, the samples that originated from 3 patients NARI-03, NARI-07, and NARI-26 shared 99–100% similarity to Gilliam, have been submitted under accession no. OK019093, OK019100, and OK019094, respectively. The nucleotide sequence OK01910 of sample NARI-33 shared 98.54% similarity with UT176 from Taiwan. The nucleotide sequences of different specimens collected from the same patient were found to be different in 2 cases. The sequence of OT isolated from enriched CSF of patient NARI-2, OK019097 clustered with Gilliam having 100% similarity, while the sequence of the blood clot (before enrichment) collected from the same individual, OK019096 shared 98.74% similarity with UT76 strain from Taiwan. Similarly, the sequence OK019098 that represented the CSF specimen of patient NARI-06 post-enrichment shared 100% similarity with the Gilliam strain; while sequence OK019099 of the enriched blood clot was identical to that of the Kato. In addition, the sequence of blood clot specimen of patient NARI-01 before enrichment (OK019092) was 99.65% similar to Gilliam, the same specimen was 99.83% similar to Kato post-enrichment (OK019095). To summarize, we could identify a total of 10 OT strains from clinical specimens belonging to 7 patients.

## Discussion

Scrub typhus was first reported in Himachal Pradesh, India, in early 1917 (Xu et al., 2017). Subsequently, many outbreaks were reported at the national borders right from World War II. Since few decades, ST is being reported from various parts of India, that spans across Jammu and Kashmir, Himachal Pradesh, Uttaranchal, Uttar Pradesh in North to Karnataka, Tamil Nadu, Kerala in south and Bihar, Assam, Meghalaya, Nagaland, Arunachal Pradesh, West Bengal in east to Maharashtra and Rajasthan in the west (Sayen and Pond, 1946; Isaac et al., 2004; Varghese et al., 2006; Bakshi et al., 2007; Kelly et al., 2009; Chrispal et al., 2010; Dass et al., 2011; Khan et al., 2012; Sinha et al., 2014; Bhargava et al., 2016; Chakraborty and Sarma, 2017; Mittal et al., 2017; Venugopal et al., 2019; Banerjee and Kulkarni, 2021). Although it is a century since first case was reported, ST still remains to be one of the underdiagnosed and neglected disease (Murhekar et al., 2016; Mittal et al., 2017). reported that approximately 60% cases with AES admitted in the Gorakhpur region were due to ST (Murhekar et al., 2016; Mittal et al., 2017; Thangaraj et al., 2018). However, a definitive diagnosis could not be made due to the lack of characteristic symptoms, limitations in diagnosis, diversity of organism along with socio-economic background, availability of healthcare facilities, and failure of the standard treatment. Hence, attempts were made to isolate and characterize the OT circulating in this region.

Various serological and molecular detection studies carried out in Gorakhpur region have reported incidence of ST (Mahajan et al., 2006; Kumar et al., 2014; Murhekar et al., 2016; Mittal et al., 2017, 2018; Vivian Thangaraj et al., 2017; Mane et al., 2019; Kamble et al., 2020; Shukla et al., 2021). Similar findings from various parts of India indicate the role of ST in cases with AES. Dominance of Kato-like, Karp-like, Gilliam, Ikada strains was reported in the three representative regions of India: North (Shimla), South (Vellore) and North-east (Shillong) (Prakash Gangwar et al., 2020). Current diagnosis of ST is based on the Weil–Felix test, ELISA, indirect immunofluorescence assay (IFA), and PCR that have limited advantages in diagnosis. While the Weil–Felix test is principally based on cross-reactivity and lacks specificity, IFA, ELISA, and PCR beat the issues of specificity and sensitivity, but the time of sample collection remains crucial for these tests. Though, ELISA is a preliminary and widely used technique to detect rickettsial infections, in our study, two sero-negative patients who seemed to have encountered infection remained underdiagnosed but were detected by PCR. One of the reasons for under diagnosis of ST is the limitation of serological tests owing to insufficient antibody production in the initial phase of infection. Thus, serological tests would be appropriate for samples collected during the convalescent phase and molecular tests would be a suitable diagnostic tool for the samples collected during the early phase of infection. However, antibiotic treatment reduces sensitivity of ST diagnosis using PCR by around 10% (Kim and Joo, 2008). Therefore, it is important to collect sample before or within 3 days of initiation of treatment with antibiotic for culturing the OT successfully and detection by PCR.

We were able to isolate and enrich OT from most of the samples that were initially detected positive by PCR except for one specimen. The possible explanation for the failure of OT-enrichment could be hospitalization of the patients at the advanced stages of the disease progression and sample collection after initiation of the antibiotic treatment.

Further, our sequencing data gave a fresh perspective to study the ST infections and circulating OT strains due to observation of two strains infecting a patient. Dual infections are reported due to the antigenic combinations or occurrence of multiple strains in humans as well as chiggers (Usha et al., 2016; Le-Viet et al., 2018; Takhampunya et al., 2018). Recently, dual infection of Kato and Kawasaki was reported by Usha et al. (2016) in Andhra Pradesh, India. Based on the species-specific serological studies, dual infections were reported in ∼1% (7 out of 663) of total blood samples included in that study; however, this was not confirmed by the genotypic characterization (Usha et al., 2016). Based on the sequencing of DNA obtained directly from the patient’s specimen, a case of dual infection has been reported from Vietnam (Le-Viet et al., 2018). The uniqueness of our study lies in the fact that we are reporting the dual infections by sequencing of DNA in (a) pre- and post-enriched specimen from an individual and (b) different specimens obtained from the same patient. Here, the possibility of one strain outgrowing other in cell culture is non-deniable. In accordance with the previous report (Varghese et al., 2013, 2015; Chunchanur et al., 2019; Kumar et al., 2019; Kannan et al., 2020; Behera et al., 2021), our study confirms the prevalence of Gilliam followed by Kato- and Karp-like strains. The prevalence of Gilliam strain was observed in four districts of UP, namely, Kushinagar, Gorakhpur, Basti, and Siddharthnagar. Dual infection of Kato was observed in the patient’s from Kushinagar and Basti, while a patient from Gorakhpur was found to be dually infected with strain UT76. We would highlight that the choice of the diagnostic tests should be made depending on the phase of infection, i.e., in accordance to the onset of symptoms. As there is a difference in virulence in OT strains, severity of disease progression may depend on the infecting strain(s). There are very few reports of dual infections in ST. Moreover, enrichment of the clinical specimens in suspected ST infections is not a routine practice in India. This makes our study unique and important, thereby, highlighting the limitations of current diagnostic practices. Owing to variable doubling time and slow growth of OT, enrichment of specimens is of very little use in diagnosis and treatment. Yet, this study is a successful attempt to explore an avenue to study diversity of circulating OT strains and is a pioneering work in India.

## Data Availability Statement

The datasets presented in this study can be found in online repositories. The names of the repository/repositories and accession number(s) can be found below: https://www.ncbi.nlm.nih.gov/genbank/, OK019092 to OK019101.

## Ethics Statement

The studies involving human participants were reviewed and approved by the Ethics Committee, National AIDS Research Institute. Written informed consent to participate in this study was provided by the participants’ legal guardian/next of kin.

## Author Contributions

NN generated and analyzed the data, and drafted the manuscript. DD assisted in designing the study, collected clinical specimens, and standardized protocols for culturing *Orientia*. AB standardized protocols for culturing and PCR of *Orientia*, additionally contributed to the manuscript writing. KZ carried out serological assays. MM and MAM recruited pediatric and adult patients, respectively, and collected samples. SK conceptualized and guided the study, data analysis, and manuscript preparation. All authors contributed to the article and approved the submitted version.

## Conflict of Interest

The authors declare that the research was conducted in the absence of any commercial or financial relationships that could be construed as a potential conflict of interest. The reviewer SK declared a shared affiliation with several of the authors NN, DD, AB, KZ, and SK, to the handling editor at the time of review.

## Publisher’s Note

All claims expressed in this article are solely those of the authors and do not necessarily represent those of their affiliated organizations, or those of the publisher, the editors and the reviewers. Any product that may be evaluated in this article, or claim that may be made by its manufacturer, is not guaranteed or endorsed by the publisher.
